# Effects of Supplemented Resveratrol on In Vitro Ruminal Fermentation and Growth Performance of Hanwoo Calves

**DOI:** 10.3390/ani12233420

**Published:** 2022-12-05

**Authors:** Chae Hwa Ryu, Byeong Hyeon Kim, Seul Lee, Han Tae Bang, Youl Chang Baek

**Affiliations:** Rural Development Administration, Animal Nutrition and Physiology Division, National Institute of Animal Science, Wanju 55365, Republic of Korea

**Keywords:** growth performance, Hanwoo calves, resveratrol, ruminal fermentation

## Abstract

**Simple Summary:**

Recently, probiotics and plant extracts have been used as alternatives to antibiotics in livestock systems. Extracts of various plants have been shown to enhance digestion, increase intestinal immunity, and affect nutrient utilization and metabolism in ruminants. However, the physiological effects of plant extracts are diverse and complex, and the exact underlying mechanism has not yet been elucidated. Resveratrol is a polyphenolic substance produced when plants are exposed to adverse environments, such as fungi and pests. It has antitoxic properties that constitute plant self-defense and antioxidant properties that inhibit fatty acid oxidation. Although the metabolism of resveratrol in the rumen is unknown, resveratrol and its metabolites may play an important role in manipulating rumen bacteria. We show that feeding resveratrol to ruminants affects their digestion and immunity. The health and immunity of young ruminants can affect disease pathogenesis and long-term fattening. We believe that healthy calves could grow stably without losing energy due to disease, which would improve their growth and boost their immunity.

**Abstract:**

We investigated the effects of resveratrol supplementation on in vitro ruminal fermentation and growth performance of Hanwoo calves. Treatment with three resveratrol concentrations (0%, 0.1%, 0.3%, and 0.5%) was used for in vitro ruminal fermentation. Resveratrol concentrations and pH of rumen fluid were negatively correlated (*p* < 0.05); therefore, total gas production, total volatile fatty acids, and acetate, propionate, and butyrate levels were significantly higher in the treatments than in the control at all time periods (*p* < 0.05). The appropriate resveratrol concentration that could be added without negative effects on the rumen was 0.3%. In farm experiments, we divided 14 Korean cattle calves into control (C) and 0.3% resveratrol (T) groups. There were no significant differences in the daily weight gain, feed conversion, final body weight, body length, withers height, and height at hip cross in the resveratrol-fed Hanwoo calves. Immunoglobulin G level was significantly higher in the treatment than in the control (*p* < 0.05), but IgA and IgM levels did not differ. Supplemental feeding of resveratrol is beneficial to in vitro ruminal fermentation, but it is important to supplement it at 0.3%. Furthermore, resveratrol affects calf immunoglobulin G.

## 1. Introduction

In livestock production systems, antibiotics are administered to animals to prevent disease and metabolic disorders, and to improve feed efficiency [1]. However, in view of the risk of residual antibiotics in milk and meat and their effects on human health, the use of antibiotics in animal feeds was prohibited in the European Union in 2006 [2]. Thus, alternative additives that would improve the efficiency of nutrient use in the rumen are sought for use in the livestock industry. Plant extracts and probiotics are promising alternatives to antibiotics [3]. Extracts of various plants have been shown to affect nutrient utilization and metabolism in ruminants by enhancing appetite and digestion, increasing intestinal immunity, and conferring antibacterial and antiviral activities [4,5,6]. The physiological activities of these plant extracts are varied and complex, and their exact mechanisms have not yet been elucidated [7].

Resveratrol (3,4′,5-trihydroxy-trans-stilbene) is a polyphenolic phytoalexin produced by plants under adverse environments, such as fungi and pests [8]. It is mainly found in grapes, peanuts, pines, mulberries, and dried persimmons [9,10]. Resveratrol normalizes oxidative glutathione reductase and bone marrow peroxidase activity by promoting the expression of enzymes involved in intracellular antioxidant processes [8]. It also inhibits H_2_O_2_ production [8]. In addition, resveratrol possesses antitoxic properties that constitute the plants’ self-defense and antioxidant properties, which inhibit fatty acid oxidation [11,12]. Resveratrol also enhances anti-inflammatory, anticancer, antiviral, antiaging, cardiovascular protection, and neuroprotective effects [13,14,15,16].

Oral administration of resveratrol has recently been reported to affect ruminal fermentation in ewes [16]. Although ruminal metabolism of resveratrol remains unknown, resveratrol and its metabolites may be important in manipulating rumen bacteria [17]. Some prior studies suggest that feeding resveratrol to ruminants affects digestion and immunity [18]. Therefore, the immunity of calves affects weaning age and carcass body weight which affects the farm’s profitability [19,20].

Therefore, we surmised that the supplementation of resveratrol would help ruminal fermentation and improve the immunity and growth of calves. However, few studies evaluating the effect of resveratrol on performance, ruminal fermentation, and immunity in Hanwoo steers have been conducted. The aim of this study was to evaluate the effect of the supplementation of resveratrol on performance, ruminal fermentation, and immunity in Hanwoo calves.

## 2. Materials and Methods

### 2.1. Chemical Analysis

This study used forage (Timothy grass) and calf concentrates (Onegiwoo, Woosung Feed Co., Ltd., Nonsan, Republic of Korea). All samples were dried at 60 °C for 48 h and ground in a cyclone mill (Foss, Hillerød, Denmark), equipped with a 1 mm screen. Dry matter (930.15), acid detergent fiber (973.18), ash (942.05), and ether extract (EE; 2003.05) were analyzed as described by Horwitz and Latimer [21]. The neutral detergent fiber (NDF) was analyzed using a heat-stable amylase and expressed inclusive of residual ash (aNDF) [22]. The levels of acid detergent-insoluble crude protein (CP) and neutral detergent-insoluble CP (NDICP) in samples were quantitated according to Licitra et al. [23]. Non-fiber carbohydrates (NFC) were estimated as 100–ash–EE–CP–(aNDF–NDICP) based on the guidelines provided by the National Academies of Science, Engineering, and Medicine (NASEM) [24]. The constituents of the experimental feed are described in Table 1.

### 2.2. Extraction of Resveratrol

The method used for extraction of resveratrol, the test substance in this study, was as follows [25]. A total of 1 kg dried mulberry was crushed to a size 3 mm or less and mixed with 1000 mL of ethanol in the dark. The mixture was then kept for 72 h and the extraction was performed at room temperature. The extract was filtered, and the residue was extracted again two more times in the same way. All the obtained filtrates were pooled, concentrated under reduced pressure at 40 °C, and then freeze-dried at −50 °C.

### 2.3. In Vitro Ruminal Fermentation

Resveratrol was used at the following three concentrations for in vitro ruminal fermentation: 0% (control), 0.1% (Treatment 1, T1), 0.3% (Treatment 2, T2), and 0.5% (Treatment 3, T3). Rumen fluid was collected before morning feeding from a 2-cannulated Hanwoo steer (655 ± 70.7 kg). The steers were placed individually in a metabolic pen and were fed 2 kg forage and 5 kg commercial concentrate twice a day (08:00 and 17:00). Fresh water and mineral blocks were available *ad libitum* throughout the experiment. The rumen fluid was filtered through four layers of cheesecloth into a flask previously filled with O_2_-free CO_2_ at 39 °C. Subsequently, 1 g of each diet was mixed with 50 mL of buffered rumen fluid in serum bottles whilst gassing with O_2_-free CO_2_. The filtered rumen fluid was mixed with McDougall’s buffer [26] at a ratio of 1:4 (rumen fluid:buffer). The buffer (pH 6.5) contained 9.8 g of NaHCO_3_, 4.62 g of Na_2_HPO_4_·2H_2_O, 0.57 g KCl, 0.47 g NaCl, 0.12 g MgSO_4_·7H_2_O, and 4 g CaCl_2_ per liter of distilled water. All treatments were performed in triplicate. The procedure was performed according to the method described by Tilley and Terry [27].

Total gas production was measured based on displacement in a glass syringe at each sampling time. The bottles were uncapped, and pH was measured with a pH meter (S20 Seven Easy™, Mettler-Toledo, Columbus, OH, USA). The concentration of ammonia nitrogen was determined as described by Chaney and Marbach [28]. Briefly, 0.02 mL of culture supernatant was mixed with 1 mL of phenol color regent and 1 mL of alkali-hypochlorite reagent and then incubated at 37 °C in a water bath for 15 min. Optical density at 630 nm was measured using a spectrophotometer (Optizen UV2120, Mecasis, Republic of Korea). The concentration of volatile fatty acids was determined as described by Erwin et al. [29]. Briefly, 1 mL of the rumen fluid supernatant was mixed with 0.2 mL of 25% (*w*/*v*) metaphosphoric acid and held at 4 °C for 30 min. After re-centrifugation of the mixture at 13,000× *g* for 10 min at 4 °C, the supernatant was injected into a gas chromatograph (HP 6890, Hewlett-Packard Co., Palo Alto, CA, USA) equipped with a flame ionization detector and capillary column (Nukol fused silica capillary column 30 m × 0.25 mm × 0.25 μm (Supelco Inc., Bellefonte, PA, USA). The temperature of the oven, injector, and detector were 180, 220, and 200 °C, respectively.

### 2.4. Growth Performance and Immunity of Hanwoo Calves

Fourteen weaned male Hanwoo (Korean native beef) calves (initial weight = 74.3 ± 4.62 kg; age = 1.5 month) were used in a feeding trial to evaluate the effects of resveratrol supplementation. The trial was performed in an experimental farm facility in Nam-won city, Korea. Experimental animals having the same initial body weight were assigned to two groups (*n* = 7 for each group). The control group was not given resveratrol, whereas the treatment group was administered resveratrol at 0.3% of concentrate selected in the in vitro rumen fermentation. The animals were individually allocated and completely randomized. All calves were fed a concentrate and Timothy grass ad libitum. Fresh water and mineral blocks were available ad libitum throughout the experiment. The experiment was conducted for a total of 78 days.

Growth performance and body sizes were measured at the beginning and at the end of the experiment. Dry matter intake was estimated based on the difference between the amounts of feed offered and refused. Feed refusals were collected the next week and weighed to determine the feed intake. Feed conversion ratio was calculated by dividing the total feed intake (kg/d) by the daily body weight gain (kg/d). Body size was measured by measuring the body length, withers height, and height at hip cross. Blood samples were collected from the jugular vein in a vacuum tube containing EDTA. Blood was centrifuged (1500× *g* at 4 °C), and plasma was collected and frozen until further analysis. Immunoglobulin G (IgG), immunoglobulin M (IgM), and immunoglobulin A (IgA) were quantitated using the Bovine IgG ELISA Kit (Neobiolab, Cambridge, MA, USA).

### 2.5. Statistical Analysis

All data analyses were performed using the SPSS software (version 26, IBM, Armonk, NY, USA). Ruminal fermentation experiments were performed using a general linear and quadratic model, and means were separated using Duncan’s multiple range test. The effect of resveratrol on the growth performance of Hanwoo calves was analyzed using the *t*-test. A *p*-value < 0.05 was considered to indicate statistical significance.

## 3. Results

### 3.1. In Vitro Ruminal Fermentation

Ruminal fermentation was as shown in Table 2 and Table 3. The pH of rumen fluid before the start of fermentation was 6.72–6.77. The ruminal pH was in the appropriate range of 5.8–7.2 [30]; we therefore believed that it had no negative effect on ruminal fermentation. After 24 h of fermentation, the pH was significantly lower in T2 and T3 than in C (Table 2, *p* < 0.05). In addition, the pH in T3 was lower than the optimal range. pH in ruminal fluid decreased with the increase in the dose of resveratrol (*p* < 0.05).

Total gas production (Table 2) and total volatile fatty acids (Table 3) were significantly higher in T2 and T3 than in the control at all times (*p* < 0.05). Additionally, acetate and propionate levels were higher in T3 than in other groups at all times (*p* < 0.05). However, there were no significant differences in butyrate and valerate levels among the treatment groups. The acetate-to-propionate ratio (AP ratio) was significantly lower in all resveratrol treatments than in the control. In addition, the resveratrol-treated groups showed a significantly lower AP ratio than the control group in a previous study [31]. The T3 group showed the lowest values at 1.99 after 48 h of fermentation (*p* < 0.05). Thus, the increase in volatile fatty acid production caused by resveratrol supplementation is indicative of more organic matter fermentation, which causes a drop in pH. Therefore, 0.3% (T2 group) was selected as the optimal concentration that can be added without causing negative effects on the rumen.

### 3.2. Growth Performance and Immunity of Hanwoo Calves

No significant differences were noted in daily weight gain, feed efficiency, and final body weight in the resveratrol feeding experiment in Hanwoo calves (Table 4). There was no difference between the experimental groups in body length, withers height, and height at hip cross at the start and end of the experiment (Table 5). IgG level in the blood was significantly higher in the treatment groups than in the control (*p* < 0.05); however, IgA and IgM levels were not different (Table 6).

## 4. Discussion

Ruminal pH and volatile fatty acids are indicators of ruminal fermentation. In ruminal fermentation, the higher the level of resveratrol addition, the lower the rumen pH and the higher the production of volatile fatty acids. We found that resveratrol altered the rumen pH and volatile fatty acids, thereby promoting ruminal fermentation. Resveratrol affects the increase in cellulolytic bacteria, which improves the digestibility of NDF and acid detergent fiber [17]. In addition, volatile fatty acids increased with the concentration of resveratrol. Ma et al. [32] have reported similar results. Decreased rumen pH may indicate enhanced fermentation, but it is also associated with acidosis. Previous studies have shown that rumen pH in the 5.8–7.2 range is normal [30]. Therefore, the concentration of resveratrol was selected within the optimal range of rumen pH to increase the levels of volatile fatty acids in the rumen.

Resveratrol is a secondary metabolite of plants and is known to improve the growth performance of animals [18,31]. However, in the study by Zhang et al. [17], a rather low concentration of 0.04 g/bw of resveratrol was used, which did not affect the growth. Addition of 0.3% (0.09 g/bw) of resveratrol did not affect calf growth performance, but increased immunoglobulin levels. Ahmed et al. [33] showed that the IgG levels increased when resveratrol was added. We also showed a high level of IgG in the blood, similar to that in previous studies. The mechanism by which phytogenic feed additives increase IgG levels is largely unknown and should be elucidated. However, improved immunity can reduce disease incidence and affect long-term growth, especially in young livestock. The immune response is known to affect the microbial community [34]. Therefore, based on this study, it is necessary to carefully select the concentration of resveratrol, and further research on the rumen microbial community is needed.

## 5. Conclusions

While supplemental feeding of resveratrol is beneficial for ruminal fermentation by increasing volatile fatty acids, may lower ruminal pH. Therefore it is essential that only 0.3% of feeds is supplemented. Resveratrol supplementation affects the immunity of calves because of its direct effects on IgG levels. However, there was no difference in calf growth after the addition of resveratrol. Resveratrol regulates immunity and growth by influencing rumen microorganisms [34], so further experiments are necessary.

## Figures and Tables

**Table 1:** Chemical composition of basal diet.

Items	Timothy	Concentrate
Dry matter (DM), %	91.54	89.32
Crude protein (CP), % of DM	9.04	20.98
Ether extract, % of DM	0.81	3.96
Neutral detergent fiber, % of DM	73.56	32.49
Acid detergent fiber, % of DM	42.57	14.58
Non-fiber carbohydrate, % of DM	15.61	37.29
Crude ash, % of DM	4.98	9.31
Neutral detergent insoluble CP, % of DM	4.02	4.03
Acid detergent insoluble CP, % of DM	1.69	1.19

**Table 2:** Effects of supplemental resveratrol on rumen pH, total gas, and ammonia nitrogen in in vitro ruminal fermentation.

Incubation Time	Control	Treatment			SEM	*p*-Value	
	C	T1	T2	T3		Linear	Quadratic
pH							
24 h	6.20 ^a^	6.15 ^a^	6.04 ^b^	5.76 ^c^	0.044	<0.05	<0.05
48 h	6.02 ^a^	5.88 ^b^	5.86 ^b^	5.62 ^c^	0.038	<0.05	<0.05
Total gas production, mL							
24 h	112.00 ^b^	128.00 ^a^	132.67^a^	127.33 ^a^	2.061	<0.05	<0.05
48 h	130.67	130.67	134.00	140.00	1.387	0.148	0.058
Ammonia nitrogen, mg/dL							
24 h	10.13	11.39	9.91	9.47	0.354	0.274	0.363
48 h	14.45	20.17	16.62	13.55	1.129	0.152	0.206

SEM, standard error of mean. C, no resveratrol; T1, 0.1% resveratrol; T2, 0.3% resveratrol; T3, 0.5% resveratrol. ^a–c^ Means with different superscripts in same column of each group are significantly different (*p* < 0.05).

**Table 3:** Effects of supplemental resveratrol on volatile fatty acid levels in in vitro ruminal fermentation.

Incubation Time	Control	Treatment			SEM	*p*-Value	
	C	T1	T2	T3		Linear	Quadratic
Total volatile fatty acids, mM							
24 h	63.50 ^d^	68.20 ^c^	72.74 ^b^	78.79 ^a^	1.526	<0.05	<0.05
48 h	75.00 ^c^	76.16 ^bc^	82.09 ^b^	92.27 ^a^	1.879	<0.05	<0.05
Acetate, %							
24 h	54.90 ^d^	54.45 ^c^	61.24 ^b^	66.02 ^a^	0.689	<0.05	<0.05
48 h	65.45 ^b^	63.67 ^b^	68.15 ^ab^	75.22 ^a^	0.869	<0.05	<0.05
Propionate, %							
24 h	22.77 ^d^	23.49 ^c^	24.43 ^b^	26.11 ^a^	0.602	<0.05	<0.05
48 h	22.17 ^c^	23.72 ^b^	24.07 ^b^	25.76 ^a^	0.690	<0.05	<0.05
Butyrate, %							
24 h	17.26 ^a^	17.16 ^a^	17.24 ^a^	16.25 ^b^	0.212	<0.05	<0.05
48 h	16.84	17.55	17.44	17.19	0.342	0.638	0.532
Valerate, %							
24 h	5.59 ^a^	5.45 ^ab^	5.38 ^b^	4.92 ^c^	0.049	<0.05	<0.05
48 h	6.11	6.28	6.25	5.76	0.096	0.254	0.115
Acetate to propionate ratio							
24 h	2.39 ^a^	2.30 ^b^	2.17 ^c^	2.02 ^d^	0.037	<0.05	<0.05
48 h	2.47 ^a^	2.21 ^b^	2.17 ^b^	1.99 ^c^	0.048	<0.05	<0.05

SEM, standard error of mean. C, no resveratrol; T1, 0.1% resveratrol; T2, 0.3% resveratrol; T3, 0.5% resveratrol. ^a–d^ Means with different superscripts in same column of each group are significantly different (*p* < 0.05).

**Table 4:** Effects of supplemental resveratrol on the growth performance of Hanwoo calves.

Items	C	T	SEM	*p*-Value
Initial body weight, kg	74.86	72.83	3.772	0.800
Daily weight gain, kg/day	1.01	0.96	0.061	0.722
Feed intake, kg/day	4.03	4.12	0.211	0.831
Forage, kg/day	0.70	0.56	0.039	0.212
Concentrate, kg/day	3.33	3.56	0.189	0.630
Feed efficiency	0.24	0.23	0.022	0.623
Final body weight, kg	153.36	147.78	1.281	0.674

SEM, standard error of mean. C, no resveratrol; T, 0.3% resveratrol.

**Table 5:** Effects of supplemental resveratrol on the body size of Hanwoo calves.

Items	C	T	SEM	*p*-Value
Initial				
Body length, cm	78.14	77.00	1.281	0.674
Withers height, cm	80.57	79.78	0.638	0.556
Height at hip cross, cm	84.86	85.00	1.078	0.950
Final				
Body length, cm	99.51	98.16	1.705	0.707
Withers height, cm	96.40	99.29	1.506	0.359
Height at hip cross, cm	100.97	101.32	1.256	0.895

SEM, standard error of mean. C, no resveratrol; T, 0.3% resveratrol.

**Table 6:** Effects of supplemental resveratrol on the immunoglobulin levels (ng/mL) in Hanwoo calves.

Items	C	T	SEM	*p*-Value
IgG	6.48	8.36	0.401	<0.05
IgM	19.04	18.26	0.167	0.486
IgA	2.81	2.61	0.532	0.572

SEM, standard error of mean. C, no resveratrol; T, 0.3% resveratrol.

## Data Availability

The data presented in this study are available on request from the first author. The data are not publicly available due to restrictions by the research group.

## References

[1] Chantziaras I., Boyen F., Callens B., Dewulf J. (2014). Correlation between veterinary antimicrobial use and antimicrobial resistance in food-producing animals: A report on seven countries. J. Antimicrob. Chemother..

[2] European Parliament and Council Regulation (EC) no. (2003). 1831/2003 of the European Parliament and of the council of 22 September 2003 on additives for use in animal nutrition. Off. J. Eur. Union.

[3] Rhodes M.J. (1996). Physiologically-active compounds in plant foods: An overview. Proc. Nutr. Soc..

[4] Hernández F., Madrid J., García V., Orengo J., Megías M.D. (2004). Influence of two plant extracts on broilers performance, digestibility, and digestive organ size. Poult. Sci..

[5] Wang R., Li D., Bourne S.C. (1998). 2000 years of herbal medicine history help us solve problems in the year 2000. Alltech’s 14th Annual Symposium.

[6] Jamroz D., Wiliczkiewicz A., Wertelecki T., Orda J., Skorupińska J. (2005). Use of active substances of plant origin in chicken diets based on maize and locally grown cereals. Br. Poult. Sci..

[7] Wallace R.J. (2004). Antimicrobial properties of plant secondary metabolites. Proc. Nutr. Soc..

[8] Bhat K.P.L., Kosmeder J.W., Pezzuto J.M. (2001). Biological effects of resveratrol. Antioxid. Redox Signal..

[9] Jeandet P., Bessis R., Gautheron B. (1991). The production of resveratrol (3,5,4′-trihydroxystilbene) by grape berries in different developmental stages. Am. J. Enol. Vitic..

[10] Sanders T.H., McMichael R.W., Hendrix K.W. (2000). Occurrence of resveratrol in edible peanuts. J. Agric. Food Chem..

[11] Dixon R.A. (2001). Natural products and plant disease resistance. Nature.

[12] Rubiolo J.A., Vega F.V. (2008). Resveratrol protects primary rat hepatocytes against necrosis induced by reactive oxygen species. Biomed. Pharmacother..

[13] Jang A., Liu X.D., Shin M.H., Lee B.D., Lee S.K., Lee J.H., Jo C. (2008). Antioxidative potential of raw breast meat from broiler chicks fed a dietary medicinal herb extract mix. Poult. Sci..

[14] Hao H.D., He L.R. (2004). Mechanisms of cardiovascular protection by resveratrol. J. Med. Food..

[15] Li Y., Cao Z., Zhu H. (2006). Upregulation of endogenous antioxidants and phase 2 enzymes by the red wine polyphenol, resveratrol in cultured aortic smooth muscle cells leads to cytoprotection against oxidative and electrophilic stress. Pharmacol. Res..

[16] Harikumar K.B., Aggarwal B.B. (2008). Resveratrol: A multitargeted agent for age-associated chronic diseases. Cell Cycle.

[17] Zhang R., Zhang W.B., Bi Y.L., Tu Y., Ma T., Dong L.F., Du H.C., Diao Q.Y. (2019). Sanguinarine and resveratrol affected rumen fermentation parameters and bacterial community in calves. Anim. Feed Sci. Technol..

[18] Zhang W., Zhang R., Bi Y., Tu Y., Tian Z., Diao Q. (2018). Effect of resveratrol and sanguinarine on growth performance and blood biochemical indices of 2–6 months old calves. Acta Vet. Zootech. Sin..

[19] Jung H.S., Jung K.K., Jang I.S. (2009). Effect of immunoglobulin Y on growth performance and blood immunological parameters in Holstein calves. J. Anim. Sci. Technol..

[20] Kwon E.G., Park B.K., Cho Y.M., Han M.H., Choi C.Y., Lee M.S. (2007). Effects of weaning age on growth performance, feed intake, disease occurrence of Hanwoo calves and reproductive efficiency of dams. J. Anim. Sci. Technol..

[21] Horwitz W., Latimer G. (2005). Official Methods of Analysis of AOAC International.

[22] Van Soest P.J., Robertson J.B., Lewis B.A. (1991). Methods for dietary fiber, neutral detergent fiber, and nonstarch polysaccharides in relation to animal nutrition. J. Dairy Sci..

[23] Licitra G., Hernandez T.M., Van Soest P.J. (1996). Standardization of procedures for nitrogen fractionation of ruminant feeds. Anim. Feed Sci. Technol..

[24] NASEM (National Academies of Science, Engineering, and Medicine) (2001). Nutrient Requirements of Dairy Cattle.

[25] Kim D.W., Hong E.C., Ji S.Y., Lee W.S., Bang H.T., Kang H.K., Kim H.S., Kim S.H. (2015). Effects of dietary resveratrol on growth performance, blood biochemical parameter, immunoglobulin, and blood antioxidant activity in broiler chicks. Korean J. Poultry Sci..

[26] Troelsen J.E., Hanel D.J. (1966). Ruminant digestion in vitro as affected by inoculum donor, collection day, and fermentation time. Can. J. Anim. Sci..

[27] Tilley J.M.A., Terry R.A. (1963). A two-stage technique for the in vitro digestion of forage crops. Grass Forage Sci..

[28] Chaney A.L., Marbach E.P. (1962). Modified reagents for determination of urea and ammonia. Clin. Chem..

[29] Erwin E.S., Marco G.J., Emery E.M. (1961). Volatile fatty acid analyses of blood and rumen fluid by gas chromatography. J. Dairy Sci..

[30] Hiltner P., Dehority B.A. (1983). Effect of soluble carbohydrates on digestion of cellulose by pure cultures of rumen bacteria. Appl. Environ. Microbiol..

[31] Ma T., Chen D.D., Tu Y., Zhang N.F., Si B.W., Deng K.D., Diao Q.Y. (2015). Effect of dietary supplementation with resveratrol on nutrient digestibility, methanogenesis and ruminal microbial flora in sheep. J. Anim. Physiol. Anim. Nutr..

[32] Ma T., Wu W., Tu Y., Zhang N., Diao Q. (2020). Resveratrol affects in vitro ruminal fermentation, methane production and prokaryotic community composition in a time- and diet-specific manner. Microb. Biotechnol..

[33] Ahmed S.T., Hossain M.E., Kim G.M., Hwang J.A., Ji H., Yang C.J. (2013). Effects of resveratrol and essential oils on growth performance, immunity, digestibility and fecal microbial shedding in challenged piglets. Asian-Australas. J. Anim. Sci..

[34] Kim K.H., Cho E.S., Kim K.S., Kim J.E., Seol K.H., Park J.C., Kim Y.H. (2015). Investigation on changes in pig farm productivity after ban of antibiotics growth promoter in commercial mixed feed. Korean J. Agric. Sci..

